# Recent Advances in Immunotherapy for Hepatocellular Carcinoma

**DOI:** 10.3390/cancers12040775

**Published:** 2020-03-25

**Authors:** Shigeharu Nakano, Yuji Eso, Hirokazu Okada, Atsushi Takai, Ken Takahashi, Hiroshi Seno

**Affiliations:** Department of Gastroenterology and Hepatology, Graduate School of Medicine, Kyoto University, Kyoto 606-8501, Japan; shnakano@kuhp.kyoto-u.ac.jp (S.N.); yujieso@kuhp.kyoto-u.ac.jp (Y.E.); okada_hiro@kuhp.kyoto-u.ac.jp (H.O.); atsushit@kuhp.kyoto-u.ac.jp (A.T.); seno@kuhp.kyoto-u.ac.jp (H.S.)

**Keywords:** hepatocellular carcinoma, immunotherapy, immune checkpoint inhibitor, PD-1, CTLA-4, combination therapy

## Abstract

Hepatocellular carcinoma (HCC) is one of the leading causes of cancer-related death since most patients are diagnosed at advanced stage and the current systemic treatment options using molecular-targeted drugs remain unsatisfactory. However, the recent success of cancer immunotherapies has revolutionized the landscape of cancer therapy. Since HCC is characterized by metachronous multicentric occurrence, immunotherapies that induce systemic and durable responses could be an appealing treatment option. Despite the suppressive milieu of the liver and tumor immunosurveillance escape mechanisms, clinical studies of checkpoint inhibitors in patients with advanced HCC have yielded promising results. Here, we provide an update on recent advances in HCC immunotherapies. First, we describe the unique tolerogenic properties of hepatic immunity and its interaction with HCC and then review the status of already or nearly available immune checkpoint blockade-based therapies as well as other immunotherapy strategies at the preclinical or clinical trial stage.

## 1. Introduction

Hepatocellular carcinoma (HCC) is the most common type of primary liver cancer and poses a serious health problem worldwide [1]. Although various surveillance systems and treatment strategies have been developed and are recommended by guidelines, including surgical resection, radiofrequency ablation (RFA), transarterial chemoembolization (TACE), systemic therapy, and liver transplantation, the prognosis of HCC remains poor due to high levels of high intra- and extra-hepatic recurrence and metastasis [2,3]. Systemic therapies using molecular-targeted agents (MTAs) have been considered efficient and are recommended for patients with advanced-stage HCC [2,4]; however, the regimens currently available are often unsatisfactory. Therefore, a novel approach that uses a different mechanism to these conventional therapies is required to improve the prognosis of HCC.

The recent development of cancer immunotherapies using immune checkpoint inhibitors (ICIs) targeting cytotoxic T-lymphocyte-associated protein-4 (CTLA-4) and anti-programmed cell death protein-1 (PD-1) has dramatically changed the landscape of cancer therapy and was awarded the Nobel Prize in 2018. Several monoclonal antibodies (mAbs) targeting CTLA-4, PD-1, or its ligand programmed cell death-ligand 1 (PD-L1) have now been approved by the FDA for various types of cancers [5]. The liver is a tolerogenic organ [6] that is relevant to successful allograft acceptance after transplantation. Thus, the development of antitumor immunity against HCC might be speculated to be synergistically impeded by this tolerogenic nature of the liver and the immunosuppressive tumor microenvironment of HCC. However, the potential of cancer immunotherapy to induce systemic and durable antitumor responses may make it an ideal therapeutic option for HCC characterized by metachronous multicentric occurrence. Indeed, several ICI therapies targeting PD-1/PD-L1 and CTLA-4 have already demonstrated promising activity against HCC and manageable safety in clinical trials, thus have been approved by the FDA. Combination ICI-based strategies have also shown promising results, while other classes of immunotherapies have begun to emerge and are being tested in preclinical and clinical studies. 

In this review, we first provide an overview of the unique intrinsic immunotolerant environment of the liver and the immune evasion mechanisms of HCC, and then review recent advances in different immunotherapy approaches and their combinations for treating HCC. 

## 2. Tolerogenic Liver Immune Environment and HCC Immune Evasion Mechanisms

The liver is a tolerogenic organ in which a unique immune environment prevents the overactivation of the immune system to antigens derived from food and bacterial products in the portal flow [6]. Immune tolerance in the liver is induced by non-parenchymal cells. Kupffer cells (KCs) are liver-resident macrophages that play a role in pathogen clearance mediated by innate immune activation [7]. However, under physiological conditions, KCs induce tolerance by impairing T cell activation or preferentially expanding regulatory T cells (Tregs) by secreting immunosuppressive factors such as IL-10, TGF- β, and prostaglandin E2 [8,9]. Liver sinusoidal endothelial cells (LSECs), which act as antigen-presenting cells (APCs) and form a cellular barrier between the liver parenchyma and sinusoid [10], are characterized by low co-stimulatory molecule levels, high immune checkpoint molecule levels, and immunosuppressive cytokine production, all of which impede their potential for T cell activation and induce immune tolerance [11,12]. Hepatic dendritic cells (DCs) mediate the induction of T cell tolerance rather than their activation [13], presumably, as they are under the influence of IL-10 and TGF-β secreted by KCs and LSECs [14]. In addition to these non-parenchymal cells, hepatocytes also function as APCs by directly interacting with and presenting antigens to naïve T cells; however, hepatocytes predispose T cells towards tolerance because they lack co-stimulatory molecule expression [15]. Together, these immunosuppressive features of the liver might impede the development of antitumor immunity.

HCC evades host immunosurveillance via multiple mechanisms; for instance, HCC cells silence the expression of tumor antigens or antigen presentation-related molecules so that cytotoxic T cells (CTLs) cannot recognize tumor cells [16,17]. HCC cells also escape immunosurveillance by expressing immune checkpoint molecules such as PD-L1 and producing various immunoinhibitory molecules, including TGF-β, IL-10, indoleamine 2, 3-dioxygenase, arginase, and adenosine [18,19]. Immunosuppressive stromal cells are also a critical component of immune dysregulation. Myeloid-derived suppressor cells (MDSCs) are a heterogeneous population of immature myeloid cells that inhibit T cell activation via iNOS, ROS, and increased arginase activity, and induce Treg expansion by producing IL-10 and TGF-β [20]. Moreover, the frequency of MDSCs in HCC patients has been reported to correlate with tumor progression [21]. Macrophages are generally categorized as having an M1 or M2 state; M1 macrophages display an antitumor phenotype by producing high and low levels of IL-12 and IL-10, respectively, whereas M2 macrophages exhibit a tumor supportive phenotype with opposite cytokine profiles. During HCC progression, hepatic macrophages are skewed from an M1 phenotype to an M2 phenotype characteristic of tumor-associated macrophages (TAMs), which act as immune suppressor cells and support tumor growth by promoting angiogenesis and tumor invasion [22]. Tregs can also impede immune surveillance against HCC due to their immunosuppressive functions; indeed, they have been shown to densely infiltrate the tumor site in patients with HCC, with the number of intratumoral Tregs acting as an independent prognostic factor of overall survival (OS) and disease-free survival (DFS) in those patients [23]. 

Even under these immunosuppressive conditions, several studies have shown that antitumor immunity exists in patients with HCC. For instance, T cells specific for four different tumor-associated antigens (TAAs) were detected in both the tumor tissue and peripheral blood of patients with HCC, with the breadth of T cell response correlating with survival [24]. Another study found that the intratumoral density of activated CTLs in patients with HCC after resection was associated with OS and that the intratumoral balance between CTLs and Tregs was associated with OS and DFS [25]. These observations suggest that the immunogenic potential of HCC could be controlled by optimized immunotherapy.

## 3. PD-1/PD-L1 and CTLA-4-Blockade Therapies

### 3.1. Basic Immunobiology of PD-1 and CTLA-4

Immune checkpoint molecules—among which, PD-1 and CTLA-4 are the best studied—play essential roles in preventing T cell overactivation by interacting with APCs and other cell types. PD-1 is a member of the CD28 family that is expressed on activated T cells, B cells, and myeloid cells and negatively regulates the immune system. The engagement of PD-1 by its ligand PD-L1 leads to the transmission of suppressive signals into T cells and the induction of peripheral tolerance [26]. In the liver, PD-L1 is constitutively expressed on liver non-parenchymal cells such as LSECs and KCs [27]; however, PD-L1 is aberrantly expressed in various tumors, including HCC tumor cells, allowing them to escape from host immune surveillance. Indeed, it has been demonstrated that tumor PD-L1 expression is associated with HCC prognosis after curative surgical treatment, suggesting that the PD-1/PD-L1 pathway is an immune escape mechanism in HCC [19]. Another member of the CD28 family, CTLA-4, is induced on naïve T cells by antigen activation but is constitutively expressed on Tregs [28]. CTLA-4 binds to CD80 and CD86 more tightly than CD28, which provides a positive signal required for T cell activation; therefore, CTLA-4 induces peripheral tolerance by counteracting CD28-mediated costimulatory signals [28]. Importantly, the expression of CTLA-4 on Tregs depletes APCs of CD80 and CD86, leaving them with a reduced ability to prime naïve T cells [28]. The intensive study of PD-1- and CTLA-4-mediated immunosuppression culminated in the dramatic success of cancer immunotherapies [29] and many clinical trials of ICI mono- and combination therapies targeting PD-1/PD-L1 and CTLA-4 in HCC have now been conducted.

### 3.2. ICI Monotherapies Directed Against PD-1 and CTLA-4

Many clinical trials have been conducted for ICI monotherapies in HCC (Table 1) and the first to be approved by the FDA was the anti-PD-1 mAb nivolumab. A phase I/II trial of nivolumab in patients with advanced HCC (CheckMate-040) showed promising results. In the dose-expansion phase in which a total of 214 patients in 4 cohorts were enrolled, the objective response rate (ORR) was 20%, the disease control rate (DCR) was 64%, and progression free survival (PFS) was 4.1 months [30]. Since adverse events (AEs) were fairly mild [30], nivolumab was approved by the FDA in September 2017 as a second-line treatment for unresectable HCC after sorafenib failure, based on subgroup analysis in CheckMate-040 [4]. However, a phase III trial (CheckMate-459) evaluating nivolumab versus sorafenib as first-line treatments in patients with unresectable HCC revealed that the trial did not achieve statistical significance for its primary OS endpoint as per the prespecified analysis [31]. The CheckMate-9DX trial is currently evaluating adjuvant nivolumab versus a placebo in HCC patients at high risk of recurrence after curative hepatic resection or ablation.

Pembrolizumab is another anti-PD-1 mAb that was granted accelerated approval by the FDA in May 2017 for patients with unresectable or metastatic microsatellite instability-high (MSI-H) or mismatch repair deficient (dMMR) solid tumors that continued to progress after conventional treatment, based on the data from five clinical trials [32]. A phase II trial (KEYNOTE-224) revealed the potential of pembrolizumab against HCC after sorafenib failure, with an ORR of 17% with one complete response (CR), a DCR of 61%, and AEs (>grade 3) reported in 26% of patients [33]. Based on this data, pembrolizumab was granted accelerated approval by the FDA in November 2018 as a second-line treatment after sorafenib. A phase III trial (KEYNOTE-240) comparing pembrolizumab to a placebo as a second-line treatment demonstrated that pembrolizumab was associated with a longer median OS and PFS; however, these findings were not deemed statistically significant according to the prespecified statistical plan [34]. Two further phase III trials are currently ongoing: KEYNOTE-394 is evaluating pembrolizumab versus a placebo and best supportive care in Asian patients with systemically treated advanced HCC, while KEYNOTE-937 is evaluating pembrolizumab versus a placebo as an adjuvant therapy in HCC patients after curative treatment.

In addition, the anti-PD-L1 mAb Durvalumab was tested in a phase I/II trial (NCT01693562) of patients with advanced HCC who had been previously treated with sorafenib, achieving an OS rate of 10.3% in 39 patients [35]. The investigational IgG4 anti-PD-1 Ab, tislelizumab (BGB-A317), was designed to bind minimally to FcγR on macrophages in order to abrogate antibody-dependent phagocytosis, which is a potential mechanism of anti-PD-1 therapy resistance. Tislelizumab has demonstrated a good preliminary safety profile and antitumor activity in a phase I trial and a phase III trial (RATIONALE 301) of tislelizumab versus sorafenib as a first-line treatment in patients with unresectable HCC is currently underway [36].

The anti-CTLA-4 mAb, tremelimumab, has been tested in a small phase II pilot trial (NCT01008358) of HCV-infected patients with advanced HCC, demonstrating partial response (PR) and stable disease (SD) rates of 17.6 and 58.8%, respectively. Moreover, the treatment was well tolerated, and no patients needed steroids due to severe immune-related AEs (irAEs) [37].

## 4. ICI-Based Combination Therapy

Although ICI monotherapy regimens have shown benefits in some HCC patients with generally acceptable AE profiles, their response rates (approximately 20%) have been unsatisfactory, presumably due to the immunosuppressive properties of the liver and HCC tumor microenvironment. To achieve enhanced therapeutic efficacy, several types of combination strategy are currently being explored (Table 2).

### 4.1. Combination of ICIs with Other ICIs or Immunostimulatory Agents

Anti-PD-1/PD-L1 and anti-CTLA-4 mAb combination strategies have been evaluated in various types of cancers. In November 2019, the FDA granted breakthrough therapy designation for nivolumab in combination with the anti-CTLA-4 inhibitor ipilimumab for patients with advanced HCC who had previously been treated with sorafenib based on data from the phase I/II CheckMate-040 study of nivolumab plus ipilimumab [38]. The study demonstrated that nivolumab + ipilimumab achieved clinically meaningful responses and had an acceptable safety profile compared to nivolumab monotherapy (ORR: 31% and 14%, respectively), with a median OS of 22.8 months in the nivolumab + ipilimumab group. Another trial (CheckMate-9DW) evaluating nivolumab + ipilimumab versus standard care (sorafenib or lenvatinib) in patients with advanced HCC who have received no prior systemic therapy is currently ongoing.

Durvalumab + tremelimumab has been evaluated in a phase I/II study of patients with advanced HCC, with an ORR of 17.5% and 7/40 evaluable patients showing PR [39]. The combination was well tolerated and showed no unexpected safety signals; therefore, a randomized phase III HIMALAYA study is currently evaluating the efficacy and safety of the durvalumab + tremelimumab combination and durvalumab monotherapy versus sorafenib as a first-line treatment for patients with unresectable HCC and no prior systemic therapy. In January 2020, the FDA granted durvalumab + tremelimumab orphan drug designation for treating patients with HCC.

Other immune checkpoint molecules, such as LAG3 and TIM-3, can also be targeted and combined with PD-1/PD-L1 or CTLA-4 blockade. For instance, phase I basket trials are currently evaluating the dual immune checkpoint blockade of LAG-3 and PD-1 (NCT03005782) and dual TIM-3 and PD-L1 blockade (NCT03099109) in patients with HCC. In addition, combination strategies involving agonistic antibodies that target costimulatory molecules such as 4-1BB, CD40, and OX40 appear to be promising. In a preclinical study, triple combination therapies targeting 4-1BB, OX40, and PD-L1 demonstrated prolonged survival in HCC-bearing mice, providing proof of concept for this combination [44]. A phase I/II basket trial is underway to evaluate the combination of agonistic anti-OX40 Abs with nivolumab and ipilimumab in patients with HCC (NCT03241173).

### 4.2. Combination of ICI and Non-Immunological Systemic Therapies

Several clinical trials are currently investigating combinations of ICIs and molecular-targeted therapies. For instance, anti-VEGF therapy has been demonstrated to not only normalize immunosuppressive tumor vasculature but also activate DCs and decrease Tregs and MDSCs [45]. In addition, a recent study demonstrated that anti-VEGF therapy rescues effector T cells from exhaustion by downregulating the transcription factor TOX [46]. Therefore, anti-VEGF therapies utilizing multi tyrosine kinase inhibitors (lenvatinib) or anti-VEGFR monoclonal antibodies (bevacizumab) appear to be quite promising in combination with ICIs.

In July 2019, the FDA granted breakthrough therapy designation for pembrolizumab in combination with lenvatinib for the first-line treatment of patients with unresectable HCC who are amenable to locoregional treatment, based on the results of a phase Ib trial (KEYNOTE-524) [40]. Consequently, a phase III trial (LEAP-002) evaluating lenvatinib + pembrolizumab vs. lenvatinib + placebo as a first-line therapy for advanced HCC is currently ongoing [41]. Recently, the results of a phase III trial (IMbrave 150) evaluating the anti-PD-L1 mAb atezolizumab + bevacizumab versus sorafenib monotherapy for patients with unresectable HCC without prior systemic therapy were presented at ESMO Asia Congress 2019, revealing that the atezolizumab + bevacizumab combination significantly improved OS and PFS compared to sorafenib [42]. Another phase III study (IMbrave 050) is currently comparing the same combination with active surveillance in HCC patients at high risk of recurrence after curative treatment, while a phase III trial (EMERALD-2) is also evaluating the durvalumab + bevacizumab combination or durvalumab alone in the same adjuvant setting. Several phase III trials are also evaluating other combinations of ICIs and MTAs: SHR-1210 + apatinib (NCT03764293), atezolizumab + cabozantinib (NCT03755791/COSMIC-312), and sintilimab (anti-PD-1) + bevacizumab biosimilar (NCT03794440/ORIENT-32).

Chemotherapeutic drugs are generally considered to be immunosuppressive agents due to their toxicity against immune cells; however, they may also be a promising partner to ICIs as they cause immunogenic cell death, allowing the release of tumor antigens and danger-associated molecular patterns from the dead tumor and enhancing the immune response [47]. In addition, some anticancer drugs downregulate Tregs and MDSCs, further promoting tumor eradication [48]. Therefore, a phase III trial evaluating SHR-1210 (anti-PD-1 Ab) + FOLFOX4 as first-line therapy in patients with advanced HCC is currently underway (NCT03605706).

### 4.3. Combination of ICIs and Non-Immunological Locoregional Therapies

Standard locoregional therapies for HCC can trigger effector T cell responses via the release of tumor-specific antigens from dead tumor cells; therefore, the combined use of locoregional therapies such as RFA, TACE, and radiation could improve the effectiveness of immunotherapies against HCC. The combination of tremelimumab + RFA was tested in a phase I/II trial (NCT01853618) of patients with advanced HCC, with PR and SD noted in five (26%) and 12 (63%) of the 19 evaluable patients, with a median time to progression (TTP) and OS of 7.4 and 12.3 months, respectively. Moreover, pathological evaluation revealed that the accumulation of intratumoral CD8+ T cells in patients had a clinical benefit [43]. TACE has been suggested to exert immunostimulatory effects as the number of α-fetoprotein (AFP)-specific T cells was observed to increase after TACE [49]. Therefore, a phase III trial (EMERALD-1) is currently evaluating TACE in combination with durvalumab and bevacizumab in patients with multiple HCCs (NCT03778957). Radiation with dual checkpoint blockade reportedly induces optimal responses in melanoma, with a previous preclinical study of melanoma demonstrating that anti-CTLA-4 increases the CTL:Treg ratio while anti-PD-L1 rescues T cell exhaustion. Moreover, radiation expanded the T cell receptor (TCR) repertoire, thereby enhancing the antitumor activity of dual checkpoint blockade [50]. Thus, these results provide proof of concept for combining ICIs and radiation to treat HCC and phase II trials are currently underway to evaluate pembrolizumab in combination with stereotactic body radiation therapy (SBRT) (NCT03316872) or Y90 (NCT03099564), and nivolumab with Y90 (NCT03033446) to treat HCC.

## 5. Exploring ICI Biomarkers

Considering the success of ICIs, it is necessary to identify predictive biomarkers for patients that will respond better to ICIs, particularly since PD-L1 expression on tumor cells does not correlate with the response to anti-PD1 therapy in patients with HCC [30]. MSI, the result of dMMR, was the first predictive biomarker for PD-1 inhibitors to be approved by the FDA [51]. MSI-H colon cancers display favorable responses to ICIs; however, MSI-H appears to be a rare event in HCC [52]. Recently, next-generation sequencing has identified Wnt/CTNNB1 mutations as possible biomarkers for predicting ICI resistance in patients with advanced HCC; however, next-generation sequencing is too complex and costly to use in clinical practice [53]. Therefore, the development of clinically and economically feasible biomarkers is a crucial yet unmet requirement in this field.

## 6. Non-ICI Immunotherapies

While ICIs release the brake on cancer immunity to unleash dysfunctional antitumor CTLs, there are other “active” immunotherapies that accelerate cancer immunity, such as cancer vaccines, oncolytic virotherapy, and cell-based therapy.

### 6.1. Cancer Vaccines

The two main cancer vaccine strategies are DC vaccines and peptide vaccines. DCs are potent APCs that can promote tumor-specific T cell responses. In DC vaccines, DCs are loaded with tumor antigens ex vivo and administered to patients as a cellular vaccine. In a preclinical mouse model, DC vaccines pulsed with tumor cell lysate effectively eradicated tumors and displayed histological evidence of intratumoral lymphocyte infiltration [54]. Unfortunately, clinical trials using DCs pulsed with tumor antigen peptides [55] or tumor cell lysate [56,57] have only demonstrated marginal activity in patients with advanced HCC thus far.

Peptide vaccines for HCC utilize shared TAAs, including AFP, glypican-3 (GPC3), and telomerase reverse transcriptase (TERT). A phase I trial of an AFP-derived peptide vaccine in 15 patients with HCC found that the vaccine was well tolerated, with CR in one patient (AFP-specific CTL response) and SD in eight patients [58]. GPC3 is another antigen that is highly expressed in HCC. In a phase I trial of 33 patients, the GPC3 peptide vaccine was well tolerated with one patient showing PR and 19 showing SD. Importantly, the GPC3 peptide vaccine induced a GPC3-specific CTL response which correlated with OS. [59]. The same group later demonstrated that PD-1 blockade augmented the efficacy of the GPC3 vaccine by increasing the number of vaccine-induced CTLs [60]. A phase II trial of a TERT-derived peptide vaccine (GV1001) in combination with low dose cyclophosphamide showed no effective antitumor response or prolonged TTP [61]. Overall, low-level clinical responses have been observed for DC- and TAA-based peptide vaccines so far; therefore, further trials should examine their combination with immunotherapy.

Neoantigen vaccines are a new cancer vaccine strategy that utilizes tumor neoantigens, which are the products of non-synonymous tumor-specific mutations and are expected to be an ideal therapeutic vaccine as they can achieve a full personalization. First, tumor mutations are analyzed by next-generation sequencing and then candidate neoantigen peptides are predicted on the basis of HLA-binding algorithms [62]. Results from phase I clinical trials testing neoantigen vaccine in advanced melanoma are quite encouraging [63,64]. The cancer vaccine development for hepatocellular carcinoma (HEPAVAC) project, which aims to produce “off-the-shelf” shared antigen-based vaccines for HCC, also includes the actively personalized vaccine (APVAC) protocol based on patient-specific neoantigens [65].

### 6.2. Oncolytic Virotherapy

Oncolytic virotherapy is a novel approach for cancer immunotherapy [66] that utilizes JX-594 (also known as Pexa-Vec), a vaccinia virus designed to preferentially replicate in and lyse tumor cells, thereby causing the release of antigens from the dead tumor cells and triggering antitumor immunity. This antitumor immunity can be further stimulated by inserting the human granulocyte-macrophage colony stimulating factor transgene into JX-594 [67], with a phase I trial showing that JX-594 has a good safety profile in patients with primary or metastatic liver cancer [68]. A randomized phase II trial has also been conducted to evaluate the safety and antitumor efficacy of JX-594 in patients with advanced HCC, finding that intratumoral JX-594 injection was well tolerated at both low and high doses. Moreover, tumor regression was observed in injected and non-injected tumors, with one CR and three PRs, and OS was significantly longer in patients that received the high dose than the low dose (median 14.1 and 6.7 months, respectively) [69]. A randomized open label phase III trial comparing sorafenib alone and JX-594 + sorafenib in patients with advanced HCC is currently underway (NCT02562755).

### 6.3. Cell-Based Immunotherapy

Cell-based immunotherapy, also known as adoptive cell transfer (ACT), is also a promising strategy that has been explored extensively. For HCC, cytokine-induced killer cells (CIKs), TCR-engineered T cells, and chimeric antigen receptor T cells (CAR-T) are the major strategies. CIKs are a mixture of heterogeneous immune cells generated by the ex vivo expansion of peripheral blood mononuclear cells in the presence of IL-2, IFN-γ, and anti-CD3 mAbs. CIKs consist of NKT cells, NK cells, and CTLs [70] and display strong cytolytic activity against tumor cells independently of MHC restriction [70]. A randomized phase II trial in treatment-naïve patients with HCC demonstrated that CIK therapy prolonged OS and PFS [71], while a multicenter open-label randomized phase III trial in patients with HCC after curative treatment demonstrated that CIK therapy prolonged recurrence-free survival and OS [72]. 

TCR-engineered T cells are generated by integrating cloned tumor antigen-specific TCR into T cells, circumventing the technical difficulties of TIL therapy wherein TILs must be isolated from tumor tissue and expanded ex vivo before being infused back into patients. In mouse models, TCR-engineered T cells recognizing AFP and GPC3 have been reported to control liver tumor growth [73,74], while phase I trials are currently evaluating genetically modified T cells expressing AFP-specific TCRs in patients with advanced HCC (NCT03132792) and autologous TCR-engineered T cell therapy targeting MAGEA1 in solid tumors such as HCC (NCT03441100).

The essential structure of CARs consists of an extracellular single-chain antibody domain that recognizes tumor antigens and an intracellular domain that transmits activation and proliferation signals into cells [75]. Antigen recognition allows CAR-T cells to eliminate cancer cells in an MHC restriction-independent manner, thus solving the problem of tumor immune escape via MHC downregulation [75]. In xenograft mouse models, CAR-T cells targeting GPC3 have been shown to eradicate GPC3-positive HCC [76], while a phase I trial of anti-GPC3 CAR-T cells with or without lymphodepletion treatment has been conducted in six patients with relapsed or refractory GPC3 positive HCC. PR and SD were observed in one and three patients, respectively, with no dose-limiting toxicity identified and only one serious AE of grade 3 fever was reported [77]. In addition, early clinical trials are currently examining CAR-T cells targeting AFP (NCT03349255), MUC-1 (NCT03198546), and EpCAM (NCT03013712).

## 7. Conclusions

HCC is a serious global health problem because current regimens have limited efficacy in HCC patients, particularly at an advanced disease stage. Cancer immunotherapy has been a significant breakthrough in cancer treatment in recent years and there has been growing interest regarding its application in HCC. As reviewed here, several classes of immunotherapy have emerged for HCC—among which, ICIs targeting PD-1/PD-L1 and CTLA4 hold the greatest promise. However, many studies evaluating ICI-based therapies and other therapeutic strategies are in progress. There are positive and negative factors that should be taken into account for developing successful immunotherapy for HCC (Table 3). Most importantly, it should be designed to counteract the unique immunosuppressive environment of the liver itself in addition to HCC. Therefore, a deeper understanding of the mechanisms underlying HCC immunology will allow the rational design of optimal therapies that coordinate the activation of both innate and adaptive immunity. Research efforts should also be directed toward identifying predictive biomarkers to avoid inappropriate treatment or overtreatment, particularly since current immunotherapies can display limited efficacy in a minority of patients, serious irAEs, and high financial cost.

## Figures and Tables

**Table 1:** Summary of clinical trials of ICI monotherapy for HCC.

Trial identifier	Target	Drugs	Phase	N	Patient Group	ORR	DCR	PFS (Median,mo)	OS (Median,mo)
NCT01658878 (CheckMate040) [30]	PD-1	Nivolumab	I/II	214*	Naive/Pre-treated	20.0%	64.0%	4	NR
NCT02576509 (CheckMate459) [31]	PD-1	Nivolumab vs. Sorafenib	III	743	Naïve	15% vs. 7%	N/A	3.7 vs. 3.8	16.4 vs. 14.7
NCT03383458 (CheckMate 9DX)	PD-1	Nivolumab vs. Placebo	III	530	Adjuvant	N/A	N/A	N/A	N/A
NCT02702414 (KEYNOTE-224) [33]	PD-1	Pembrolizumab	II	104	Pre-treated	17.0%	61.0%	4.9	12.9
NCT02702401 (KEYNOTE-240) [34]	PD-1	Pembrolizumab vs. Placebo	III	413	Pre-treated	18.3% vs. 4.4%	62.2% vs. 53.3%	3.0 vs. 2.8	13.9 vs. 10.6
NCT03062358 (KEYNOTE-394)	PD-1	Pembrolizumab vs. Placebo	III	N/A	Pre-treated	N/A	N/A	N/A	N/A
NCT03867084 (KEYNOTE-937)	PD-1	Pembrolizumab vs. Placebo	III	N/A	Adjuvant	N/A	N/A	N/A	N/A
NCT03412773 (RATIONALE-301) [36]	PD-1	Tislelizumab vs. Sorafenib	III	N/A	Naïve	N/A	N/A	N/A	N/A
NCT01693562 [35]	PD-L1	Durvalumab	I/II	39	Pre-treated	10.3%	33.3%	NA	13.2
NCT01008358 [37]	CTLA-4	Tremelimumab	II	20	Pre-treated	17.6%	76.4%	6.48	8.2

N, number of patients; N/A; not available; NR, not reached; * dose-expansion phase.

**Table 2:** Summary of clinical trials of ICI combination therapy for HCC.

Trial Identifier	Target	Drugs	Phase	N	Patient Group	ORR	DCR	PFS (Median, mo)	OS (Median, mo)
***ICI + ICI***									
NCT01658878 (CheckMate040) [38]	PD-1 + CTLA-4	Nivolumab + Ipilumumab	II	148	Pre-treated	31% (5%CR)	49.0%	NA	22.8 (arm A)
NCT04039607 (CheckMate 9DW)	PD-1 + CTLA-4	Nivolumab + Ipilimumab vs. Sorafenib/lenvatinib	III	1084	Naïve	N/A	N/A	N/A	N/A
NCT02519348 [39]	PD-L1 + CTLA-4	Durvalumab + Tremelimumab	I/II	40	Naive/Pre-treated	15.0%	57.5% at 4 mo	NA	NA
NCT03298451 (HIMALAYA)	PD-L1 + CTLA-4	Durvalumab + Tremelimumab vs. Sorafenib	III	1310	Naïve	N/A	N/A	N/A	N/A
***ICI + MTA***									
NCT03006926 (KEYNOTE-524) [40]	PD-1 + MTA	Pembrolizumab + Lenvatinib	Ib	30	Naive	36.7%	90.0%	9.7 (TTP)	14.6
NCT03713593 (LEAP-002) [41]	PD-1 + MTA	Pembrolizumab + Lenvatinib vs. Lenvatinib	III	750	Naïve	N/A	N/A	N/A	N/A
NCT03434379 (IMbrave150) [42]	PD-L1 + MTA	Atezolizumab + Bevacizumab vs. Sorafenib	III	501	Naive	33% vs. 13%	NA	6.8 vs.4.3	NR vs. 13.2
NCT04102098 (IMbrave050)	PD-L1 + MTA	Atezolizumab + Bevacizumab vs. Placebo	III	662	Adjuvant	N/A	N/A	N/A	N/A
NCT03847428 (EMERALD-2)	PD-L1 + MTA	Durvalumab + Bevacizumab vs. Bevacizumab	III	888	Adjuvant	N/A	N/A	N/A	N/A
NCT03764293	PD-1 + MTA	SHR-1210 + Apatinib vs. Sorafenib	III	510	Naive	N/A	N/A	N/A	N/A
NCT03755791 (COSMIC-312)	PD-L1 + MTA	Atezolizumab + Cabozantinib vs. Sorafenib	III	740	Naive	N/A	N/A	N/A	N/A
NCT03794440 (ORIENT-32)	PD-1 + MTA	Sintilimab + Bevacizumab biosimilar vs. Sorafenib	III	566	Naive	N/A	N/A	N/A	N/A
***ICI + Chemo***									
NCT03605706	PD-1 + chemotherapeutic agents	SHR-1210 + FOLFOX4 regimen vs. Sorafenib or FOLFOX4 regimen	III	448	Naive	N/A	N/A	N/A	N/A
***ICI + ablation***									
NCT01853618 [43]	CTLA-4	Tremelimumab + ablation	I/II	32	Advanced	26.0%	85.0%	7.4 (TTP)	12.3
***ICI + TACE***									
NCT03778957 (EMERALD-1)	PD-L1	Durvalumab + TACE or Durvalumab + Bevacizumab +TACE vs. TACE alone	III	600	Locoregional(Naïve)	N/A	N/A	N/A	N/A
***ICI + Radiation***									
NCT03316872	PD-1 + Radiation	Pembrolizumab + Radiation (SBRT)	II	30	Pre-treated	N/A	N/A	N/A	N/A
NCT03099564	PD-1 + radioembolization	Pembrolizumab + Y90 radioembolization	I	30	Locoregional	N/A	N/A	N/A	N/A
NCT03033446	PD-1 + radioembolization	Nivolumab + Y90 radioembolization	II	40	Advanced	N/A	N/A	N/A	N/A

MTA, molecular-targeted agent; SBRT, stereotactic body radiotherapy; TACE, transarterial chemoembolization; Y90, Yttrium-90; Apatinib, VEGFR2 inhibitor; Cabozantinib, multi kinase inhibitors of MET, VEGFR2, FLT3, c-KIT and RET; N, number of patients; N/A, not available; NR, not reached.

**Table 3:** Positive and negative factors for developing successful immunotherapy for HCC.

Positive factors		Negative Factors	
1	Immunotherapy can induce not only systemic but also durable responses by immunological memory, both of which are advantageous for controlling HCC that is characterized by metachronous multicentric occurrence.	1	Paucity of biomarkers predicting responders and non-responders.
2	The presence of tumor-infiltrating lymphocytes (TILs) in HCC suggests the potential of hosts to induce endogenous tumor immunity.	2	Tolerogenic nature of hepatic immunity and immunosuppressive tumor microenvironment of HCC.
3	Several ICIs have already demonstrated manageable safety and promising activity in clinical trials.	3	Response rates of ICI monotherapy are not satisfactory.
